# Towards Polycaprolactone-Based Scaffolds for Alveolar Bone Tissue Engineering: A Biomimetic Approach in a 3D Printing Technique

**DOI:** 10.3390/ijms242216180

**Published:** 2023-11-10

**Authors:** Krzysztof Stafin, Paweł Śliwa, Marek Piątkowski

**Affiliations:** 1 Department of Organic Chemistry and Technology, Faculty of Chemical Engineering and Technology, Cracow University of Technology, ul. Warszawska 24, PL 31-155 Kraków, Poland; krzysztof.stafin@jezuici.pl (K.S.); pawel.sliwa@pk.edu.pl (P.Ś.); 2 Department of Biotechnology and Physical Chemistry, Faculty of Chemical Engineering and Technology, Cracow University of Technology, ul. Warszawska 24, PL 31-155 Kraków, Poland

**Keywords:** bone tissue engineering, alveolar bone, biomineralisation, biomimetic ossification, fused filament fabrication, 3D-printed PCL-based scaffold, hydroxyapatite, collagen, chitosan

## Abstract

The alveolar bone is a unique type of bone, and the goal of bone tissue engineering (BTE) is to develop methods to facilitate its regeneration. Currently, an emerging trend involves the fabrication of polycaprolactone (PCL)-based scaffolds using a three-dimensional (3D) printing technique to enhance an osteoconductive architecture. These scaffolds are further modified with hydroxyapatite (HA), type I collagen (CGI), or chitosan (CS) to impart high osteoinductive potential. In conjunction with cell therapy, these scaffolds may serve as an appealing alternative to bone autografts. This review discusses research gaps in the designing of 3D-printed PCL-based scaffolds from a biomimetic perspective. The article begins with a systematic analysis of biological mineralisation (biomineralisation) and ossification to optimise the scaffold’s structural, mechanical, degradation, and surface properties. This scaffold-designing strategy lays the groundwork for developing a research pathway that spans fundamental principles such as molecular dynamics (MD) simulations and fabrication techniques. Ultimately, this paves the way for systematic in vitro and in vivo studies, leading to potential clinical applications.

## 1. Introduction

### 1.1. Bone Tissue Engineering (BTE)

The alveolar bone, which secures teeth in the jaw, can undergo resorption following tooth extraction due to the lack of mechanical load [1,2]. The regeneration of alveolar bone, as opposed to skeletal bone, poses unique clinical challenges. While autologous bone grafts are considered the “gold standard” for significant alveolar bone defects [3], they come with drawbacks such as donor site morbidity, deformity, potential infection, and the risk of graft rejection. Consequently, research in material science and tissue engineering is focused on finding suitable materials and their fabrication for the replacement and reconstruction of alveolar bone [4]. BTE focuses on crucial processes involving cell growth and the complex structure of human bone at both microscopic (biomineralisation) and macroscopic (ossification) scales [5].

Numerous methods have been explored to halt the resorption of the alveolar process [6], and research on various strategies for reconstructing alveolar bone defects is rapidly expanding. However, the included studies exhibit a high degree of heterogeneity [7], necessitating the establishment of a unified standard for bone regeneration in the alveolar process [8]. The advancement of medical science, particularly in implantology, relies on the development of a robust research methodology that enables a legitimate transition from correlational conclusions to causative ones, thereby minimising potential errors before clinical trials. As an interdisciplinary field, BTE combines osteoconductive scaffolds, osteogenic cells, growth factors, and their interrelationships within the natural microenvironment [9]. An integrated methodological approach is proposed to optimise scaffold characteristics, grounded in interdisciplinary research encompassing regenerative medicine, materials, tissue, and dental engineering. Although many phenomena in nature are exceptionally complicated and challenging to replicate, BTE employs a biomimetic approach. This approach provides unique and innovative solutions, offering one strategy to individual fields [10].

### 1.2. Fused Filament Fabrication (FFF) vs. Other Three-Dimensional (3D) Printing Techniques

Among the promising strategies in this area, the employment of scaffolds fabricated via 3D printing techniques stands out. One such technique is FFF, also known as fused deposition modelling (FDM) [11,12]. Other methods include PolyJet, selective laser sintering (SLS), digital light processing (DLP), stereolithography (SLA), and bioprinting [13]. In comparing FFF with these alternatives, it is valuable to consider various aspects such as cost, materials employed, adaptability of the technique to the structure of the model produced, and the safety associated with the technique’s use [14,15,16].

The PolyJet 3D printing technique [13] utilises photopolymerisation, utilising resin streams for construction. In contrast, SLS [17,18] uses a laser to sinter polymer powders, which facilitates the production of complex geometries that may be more difficult to realise with other methods. DLP [19] cures resins using light and can deliver rapid printing times. SLA [20] is distinguished by its high resolution and ability to fabricate intricate structures precisely. Bioprinting, a specialised subset of 3D printing technology, is engineered to construct biological structures encompassing tissues and organs [21].

While each technique mentioned above possesses distinct advantages, FFF is particularly notable for its cost-effectiveness—it is comparatively economical, enhancing its accessibility [22]. It also supports a wide selection of biocompatible materials, including various biodegradable polymers, which are increasingly relevant in BTE. Furthermore, FFF offers versatility to accommodate specific user needs, allowing for the straightforward customisation of designs without significantly impacting production costs [23]. FFF printers are generally simpler to construct than their counterparts in the 3D printing domain. For instance, the absence of laser radiation or ultraviolet (UV) light in FFF enhances user safety, positioning this technology as a viable option for industrial upscaling within BTE contexts. The inherent simplicity and flexibility of FFF technology make it an exemplary candidate for manufacturing processes aligned with the principles of lean manufacturing and rapid prototyping.

The FFF technology involves the layer-by-layer deposition of fused material to construct a 3D object. This process initiates with the generation of a 3D computer model, which is subsequently ‘sliced’ into thin cross-sections utilising specialised slicing software. This software delineates the pathways for the print head to follow and sets various printing parameters, including temperatures, speeds, and infill patterns. The print head, or extruder, is heated to a predetermined temperature, facilitating the melting of the filament. This filament, a strand of thermoplastic material typically measuring 1.75 ± 0.15 mm in diameter, is directed into the heated extruder [16]. Within the extruder, the material liquefies and is extruded through a narrow nozzle, generally 0.4 mm in diameter [24], onto the build platform. The extruder traverses the preordained paths, laying the material in successive layers. Upon completion of a layer, the build platform descends (or the extruder ascends, contingent upon the printer design), and the process iterates for the subsequent layer. The newly deposited material cools and solidifies, bonding with the layer beneath it. After the printing concludes, the final object is meticulously detached from the build platform [13].

Currently, two significant bottlenecks are identifiable within the context of BTE concerning FFF. The first involves the processing of the filament to ensure it satisfies the requisite material specifications. The second entails the fine-tuning of printing parameters to achieve a scaffold with properties that are appropriately tailored for BTE.

### 1.3. Polycaprolactone (PCL) vs. Other Polyesters

A scaffold, which imitates the bone tissue structure, can minimise the risk of rejection by the body and foster bone regeneration [25]. Three-dimensional printing enables the precise and controlled fabrication of personalised spatial structures with complex geometries, adaptable to the anatomical shapes of the alveolar bone [26,27]. One of the most promising materials is PCL [28], mainly due to a suite of properties that set it apart from other synthetic polyesters, such as polyglycolic acid (PGA), polylactic acid (PLA), and poly(lactic-co-glycolic acid) (PLGA) [29].

PGA is a bioresorbable, aliphatic polyester frequently utilised in the medical domain, especially for surgical sutures, owing to its high tensile strength and degradability [30]. PGA scaffolds have been employed in BTE on account of their mechanical attributes. Nevertheless, PGA undergoes rapid degradation and generates acidic by-products, which could affect the local cellular milieu and impede tissue regeneration.

PLA is another aliphatic polyester derived from renewable resources like cornstarch or sugarcane. Besides its widespread use in biodegradable packaging, PLA is employed in the medical field for sutures, drug delivery systems, and tissue engineering scaffolds [31]. However, similar to PGA, the degradation of PLA can lead to acidic by-products, potentially affecting the cellular environment.

PLGA is a copolymer of PLA and PGA that combines the properties of both polymers. Its versatility, evidenced by its tunable degradation rates and mechanical properties, makes it a preferred choice for drug delivery and tissue engineering. PLGA scaffolds can offer intermediate degradation rates between PLA and PGA while maintaining sufficient mechanical strength [32]. However, as with its parent polymers, the degradation by-products can influence the pH of the local environment, which might necessitate careful consideration during scaffold design and application.

In the realm of BTE, the choice between PGA, PLA, and PLGA often hinges on the specific requirements of the application, including the desired degradation rate, mechanical properties, and biological response of the surrounding tissue. PCL offers advantages that address some of the limitations inherent in the polymers mentioned above.

In BTE, one pivotal consideration is the processing properties of PCL, which include a low melting point (approx. 60 °C) [33]. Additionally, PCL’s flexibility, attributable to its low glass transition temperature (approx. −60 °C) [34], along with its thermoplasticity, renders it more amenable to being shaped into various forms when compared to the relatively more brittle PLA. Moreover, the balance between stiffness and flexibility in PCL can be modulated [10,28], enhancing its versatility. It readily blends with other polymers or ceramics to form copolymers or composites, thereby facilitating the fabrication of scaffolds with properties that are optimally tailored for bone tissue regeneration.

The second pivotal consideration is scaffold longevity within the body, referring to structural and mechanical integrity. While PGA, PLA, and PLGA present distinct advantages, they typically degrade more rapidly than PCL. The slower degradation rate of PCL [28] is particularly advantageous in BTE, as it ensures the scaffold remains intact, providing consistent mechanical support throughout the development and maturation of bone tissue.

Currently, two limitations of PCL scaffolds are noted. One involves carrying out an appropriate compatibilisation strategy to strengthen the surface to hydrophilic properties. Hydrophilisation is correlated with facilitating cell anchoring and adhering. The second involves the adaptation of the degradation rate of material to the remodelling of bone tissue. However, due to PCL’s degradation rate and surface properties, its sole use might not adequately stimulate regenerative processes in bone tissue. Therefore, the introduction of ceramics and natural polymers [35] into the 3D scaffold presents a solution since these materials have high bioactive potential.

### 1.4. Hydroxyapatite (HA), Type I Collagen (CGI), and Chitosan (CS)

The incorporation of ceramic materials into the PCL matrix permits the regulation of both degradation and resorption rates. Their bioactivity is unmatched, enhancing the interaction with bone cells. In accordance with the biomimetic approach, HA is identified as a promising material owing to its structural congruence with bone tissue. Other materials, such as β-tricalcium phosphate (β-TCP) [36], bioactive glass, and silica-based ceramics, also promote the formation of bone tissue; however, their bioactivity is somewhat inferior compared to HA. Furthermore, their biodegradation rates and biocompatibility are less optimally adaptable to specific requirements than those of HA.

In the biomimetic approach to BTE, CGI and CS are deemed appropriate materials. CGI, in conjunction with HA—the quintessential organic and inorganic components of bone tissue—plays a pivotal role in biomimetic mineralisation [37]. Together, they replicate the natural microstructural matrix, providing an optimal environment for tissue regeneration. CS, a natural polysaccharide within the glycosaminoglycans (GAGs) family, also demonstrates significant bioactive potential [38,39,40]. Renowned for its biocompatibility, capacity to promote cell growth, and ability to inhibit bacterial proliferation, CS facilitates the biomimetic mineralisation process, which involves the synthesis of mineral substances similar to those in natural bone tissue [41]. Compared to other natural polymers, CS has distinct advantages:

- It exhibits superior mechanical properties and a more streamlined purification process than silk fibroin [42].
- Its production is less costly and arises from more diverse sources than hyaluronic acid [43].
- CS surpasses bacterial cellulose in biodegradation within physiological environments and demonstrates enhanced osseointegration [44].

Utilising a 3D scaffold comprising PCL, HA, CGI, and CS can establish a biomimetic environment that triggers regenerative processes at the alveolar bone defect site [45,46]. The scaffold’s structure allows the adhesion of bone cells, providing mechanical support, while CGI and CS act as active ingredients stimulating osteoblasts’ anchoring, proliferation, and differentiation [47,48]. Through the synergy of these components, the PCL-based 3D scaffold can accelerate the regeneration process, leading to bone mineralisation at the defect site. This approach paves the way for practical BTE by establishing a biomimetic environment conducive to restoring the structure and function of bone tissue in a manner harmonious with the body’s biology, thereby leading to improved therapeutic results.

**Figure 1:**
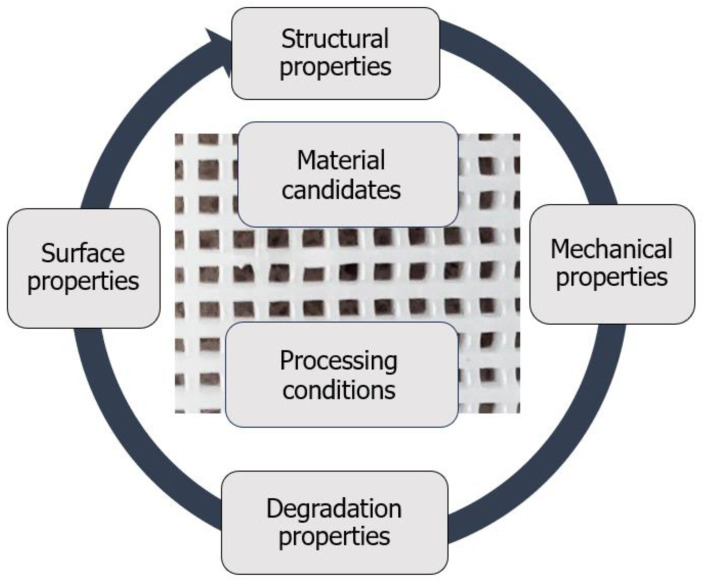
Scheme incorporating the most critical properties and parameters in the fabrication of 3D-printed PCL-based scaffolds.

This review outlines the main challenges in designing osteoconductive scaffolds with osteoinductive potential for alveolar bone engineering. Emphasis is placed on understanding processes such as biomineralisation and ossification. Through a biomimetic approach, we examine a range of properties that these scaffolds should possess (Figure 1). Given that reviewing processing conditions and structural, mechanical, degradation, and surface properties is complex and subject to debate, we focus on discussing and identifying research gaps.

## 2. Alveolar Bone

The alveolar bone is unique due to its embryonic cellular origin, specific ossification process, and connection to teeth via periodontal tissues. This bone plays a pivotal role in safely anchoring teeth, enabling them to withstand the forces generated during chewing and biting (mechanical function) [6,8]. Additionally, the alveolar bone contributes to maintaining the shape of the mouth by connecting the tooth root with surrounding bone tissue (stabilisation function) [49]. This linkage secures the tooth’s position and reduces the risk of tooth loss due to periodontal disease or other oral health issues [50].

Moreover, the alveolar bone plays a significant role in tooth development. As the teeth evolve, the bone grows and reshapes to accommodate the emerging tooth. The alveolar bone also aids in maintaining teeth alignment posteruption, regulating the movement and positioning of periodontal ligaments [51].

**Figure 2:**
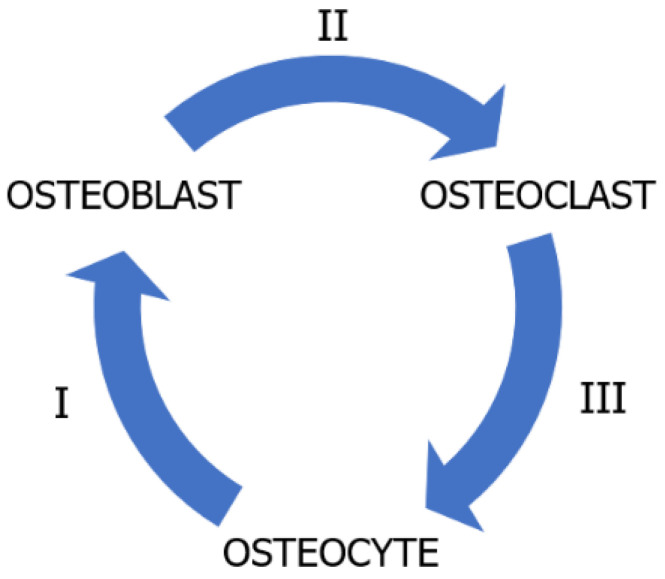
Scheme of osteocyte–osteoblast–osteoclast interactions enclosed in coupling system. (I) Osteocyte–osteoblast interactions: osteocytes possess the ability to detect mechanical loads on bone. When a bone is loaded, osteocytes convert this load into biochemical signals. Subsequently, osteocytes can release factors, such as prostaglandins and nitric oxide (NO), which modulate osteoblast activity. Through this mechanism, osteocytes promote the activation of osteoblasts to form new bone in response to loading. (II) Osteoblast–osteoclast interactions: osteoblasts produce and release factors such as RANKL (receptor activator of nuclear factor ϰ-B ligand) and M-CSF (macrophage colony-stimulating factor). RANKL binds to the RANK receptor on the surface of osteoclast precursors, leading to their differentiation into fully active osteoclasts. Additionally, osteoblasts produce osteoprotegerin (OPG), which serves as a ‘trap’ for RANKL, inhibiting osteoclast activation. The balance between RANKL and OPG in the bone microenvironment determines whether bone will be resorbed or formed. (III) Osteocyte–osteoclast interactions: osteocytes can also release RANKL, influencing the activation of osteoclasts. Furthermore, osteocytes can secrete factors that inhibit osteoclast activity in response to mechanical loading [6,55,56,57].

Understanding the unique function and structure of the alveolar bone can significantly contribute to maintaining oral homeostasis, which is a balance between the resorption and formation of bone [6]. Bone tissue homeostasis is strongly related to the balanced activities of osteoblasts and osteoclasts, which are responsible for bone formation and resorption, respectively. Moreover, osteocytes play a pivotal role in regulating both of these processes [8,52]. These cells interact through mechano-biochemical signals with each other to regulate the processes of bone formation and resorption, which is crucial for bone homeostasis (Figure 2) [6,53].

Thus, the amount of bone tissue resorbed by osteoclasts is balanced by the amount of new bone formed by osteoblasts, ensuring that the net bone mass remains constant (coupling system). However, this equilibrium is disrupted following tooth extraction or during the early healing phase of severe periodontitis, resulting in excessive osteoclastic bone resorption—a primary characteristic of jawbone disease. The ultimate consequence of the alveolar bone’s physiological remodelling is localised loss [6,53].

Therefore, all processes, including the ossification of the alveolar bone, are integral to oral health and development. This diverse jaw structure’s morphological and mechanical features necessitate innovative therapeutic approaches [54].

### 2.1. Ossification and Biomimetic Ossification

Ossification is the overarching process of bone formation. It predominantly results in the growth and repair of hard tissue at the macroscopic scale. Mimicking natural biological processes by inserting a PCL-based scaffold into an alveolar bone defect poses a significant challenge. The scaffold’s architecture is designed to provide stability and mechanical strength commensurate with bone tissue.

#### Mechanisms of Ossification

**Figure 3:**
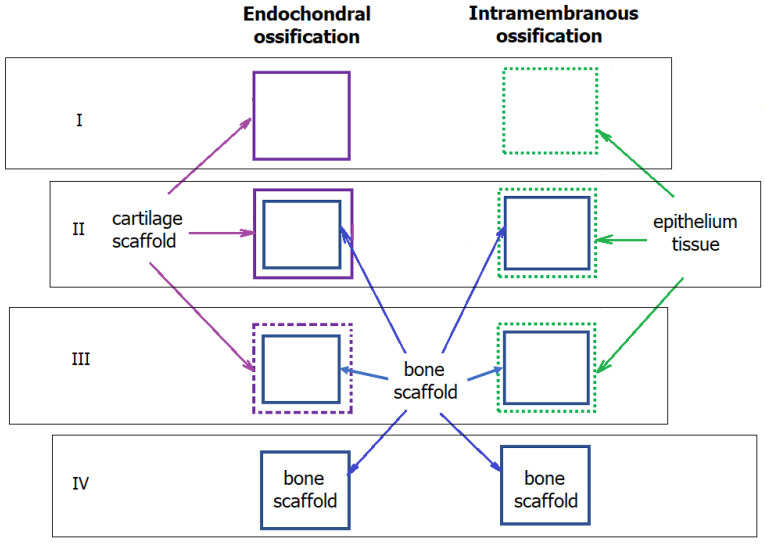
Diagram of the stages of bone ossifications. Endochondral ossification: (I) the formation of model cartilage (scaffold) rich in chondrocytes; osteoblasts migrate inside the cartilage scaffold; (II) inside the cartilage scaffold, osteoblasts produce primary bone in the form of bone trabeculae; (III) simultaneous loss of model cartilage and growth of bone tissue (chondrocytes undergo apoptosis and the cartilage matrix is degraded). Intramembranous ossification: (I) the formation of a tissue membrane rich in fibroblasts; fibroblasts differentiate into osteoblasts; (II) based on membrane tissue, osteoblasts produce primary bone in the form of bone trabeculae, which are connected to form a network; (III) a bone tissue grows in the space. For both of them: (IV) a bone is constantly transformed and adapted to the body’s needs; osteoblasts, osteoclasts, and osteocytes control remodelling.

Ossification primarily occurs through endochondral and intramembranous mechanisms [21,58]. Figure 3 offers a comparative overview of these processes. Alveolar bone ossification proceeds via the intramembranous mechanism [6,59]. From an engineering perspective, these two mechanisms exhibit several key distinctions.

Firstly, endochondral ossification occurs within a pre-existing cartilaginous scaffold, which provides inherent mechanical stability during ossification and biodegrades as the bone tissue gains enough mechanical strength [60]. In contrast, intramembranous ossification directly occurs within connective tissue, lacking the mechanical robustness provided by a cartilaginous scaffold. This aspect makes the developing intramembranous ossification more vulnerable to external forces, potentially leading to deformities in the bone architecture [61].

Secondly, the origins of osteoblasts, the cells responsible for bone formation, differ between the two processes. In endochondral ossification, osteoblasts initially form outside the cartilaginous scaffold, typically differentiating in the bone marrow [62]. These cells then migrate into the scaffold, where they proliferate and differentiate. Conversely, osteoblasts directly originate within the connective tissue in intramembranous ossification, differentiating from mesenchymal cells resembling primitive fibroblast precursors.

Consequently, the two ossification processes significantly differ in their mechanisms, particularly regarding their mechanical implications and osteoblast formation [63]. It would be intriguing to hypothesise whether introducing a scaffold could alter the biological behaviour of the healing and ossification process.

Understanding the ossification mechanism of the alveolar bone aligns with the biomimetic approach and aids in designing bone replacement material. The scaffold at the defect site should provide sufficient space for osteoblast development without creating a spatial barrier. One method to minimise this spatial barrier, thereby promoting the differentiation of osteoblasts (bone ossification) and fibroblasts (bone healing), is to enable the scaffold to transmit mechanical forces and convert them into a biochemical signal (mechanotransduction) [64].

### 2.2. Biomineralisation and Biomimetic Mineralisation

Bone mineralisation, a specific stage of ossification at the microscopic scale, involves the precipitation of calcium (Ca^2+^) and primarily monohydrogen phosphate (HPO_4_^2−^, P_i_) ions [65]. The typical biogenic mineral is hydroxyapatite (Ca_10_(PO_4_)_6_(OH)_2_, HA), an inorganic component integral to bones and teeth [66]. Biomimetic mineralisation is a strategy that seeks to emulate natural mineralisation processes to create hydroxyapatite with a crystallographic structure that resembles bone apatite [67]. For instance, Cai et al. utilised X-ray spectroscopy to analyse the resulting materials. They found that the calcium-to-phosphorus (Ca/P) ratio of the synthesised hydroxyapatite (1.65) was similar to that of natural bone tissue (1.67) [68]. While the precise mechanism of biomineralisation remains controversial and not entirely understood due to limitations and gaps in comprehending this process in vivo [69], the control of hydroxyapatite (HA) crystallisation by an organic matrix, such as type I collagen (CGI), is common to all apatite-containing hard tissues [5,70].

#### 2.2.1. Stages of Biomineralisation

**Figure 4:**
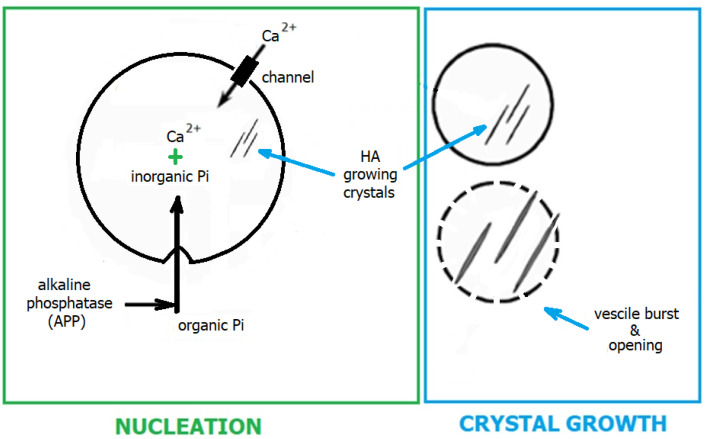
The formation of HA crystals in and out of the matrix vesicles.

From a crystallisation standpoint, biomineralisation can be divided into two stages: the nucleation of calcium phosphate (Ca_3_(PO_4_)_2_, CaP_i_) (i) and the growth of its crystals in the form of apatite (ii) (Figure 4).

**Figure 5:**
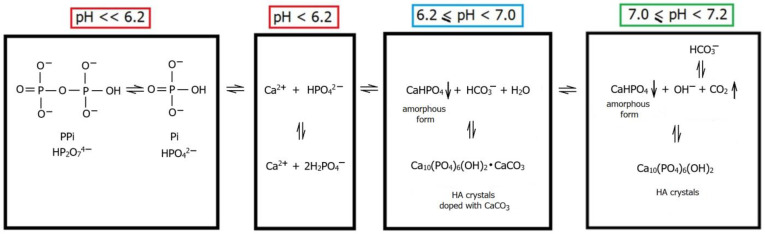
Scheme of the behaviour of Ca and P_i_ depending on pH in the matrix vesicle.

HA nucleation occurs in matrix vesicles produced by osteoblasts and released into the extracellular matrix (ECM) [71]. Ca^2+^ is transported inside the vesicle by the Ca^2+^ pump, and P_i_ is converted from organic to inorganic by alkaline phosphatase. The matrix vesicle contains CGI, the matrix for CaP_i_ precipitation. Thus, this process occurs at the interface of the inorganic–organic phase [72,73]. From a biochemical perspective, the precipitation behaviour of CaP_i_ can be influenced by critical parameters, such as pH and the concentration of individual Ca^2+^ and P_i_. Three variants are identified within the matrix vesicles, where a unique microenvironment prevails (Figure 5). In the first variant (pH equal to or lower than 6.2), biomineralisation does not occur, even if an appropriate concentration of Ca^2+^ and P_i_ ions is present in the matrix vesicles. Equilibrium is then established between the concentrations of monohydrogen phosphate (HPO_4_^2−^, P_i_) and dihydrogen phosphate (H_2_PO_4_^−^) ions, where their concentration remains constant. In addition, HPO_4_^2−^ is condensed into pyrophosphate (HP_2_O_7_^4−^, PP_i_) ions. The second variant occurs in a neutral or slightly alkaline environment (pH in the range of 7.0 to 7.2). Here, the precipitation process of Ca^2+^ and HPO_4_^2−^ occurs.

Furthermore, the bicarbonate (HCO_3_^−^) ions are enzymatically degraded to carbon dioxide (CO_2_) and the hydroxyl anion (OH^−^). It can be hypothesised that CO_2_ contributes to the rupture of the matrix vesicle, and the OH^−^ is incorporated into the apatite structure, forming HA. The third variant (pH in the range of 6.2 to 7), which is most characteristic of bone, involves HCO_3_^−^ not being fully decomposed and incorporated into the apatite structure [74]. Consequently, hydroxyapatite in bones, typically doped with bicarbonate (so-called bone apatite), is characterised by easier solubility, facilitating dynamic bone remodelling [75,76].

Crystal growth primarily occurs after the matrix vesicle opens. The standard size of the basal unit of bone apatite is 30–50 nm (length), 15–30 nm (width), and 2–10 nm (thickness) [77], exhibiting a rodlike or platelike microstructure, and is embedded in collagen fibres [78]. Bone apatite constitutes approximately 50 vol.% of the mature bone. The specific microstructural organisation of bone is age-dependent and varies among different bones and within different locations of the same bone [79]. HA crystals are deposited on collagen fibrils in intrafibrillar and extrafibrillar spaces [37,74]. HA crystal growth is regulated by noncollagenous proteins such as osteocalcin and SIBLING [80].

In addition, glycosaminoglycans (GAGs) also appear to be indirectly involved in HA crystal growth [81,82]. Many studies on bone mineralisation focus on the role of CGI and cells such as osteoblasts and osteoclasts, which may draw attention away from the functions of GAGs. Properties of GAGs, such as their ability to bind and hold water protection against mechanical stresses or interactions with proteins and minerals [83,84], may play pivotal roles in HA crystal growth. Therefore, further research is necessary to understand these processes better.

GAGs, soft and hard tissue ECM components, are highly anionic straight-chain polysaccharides composed of repeating disaccharide units. Each unit consists of amino sugar and uronic acid. GAGs in bones play a vital role in transmitting mechanical forces. Their structure enables them to absorb impact energy, reduce the risk of injury, and contribute to bone toughness by retaining bound water in a bone mineral matrix [85]. The GAGs align specifically with the CGI, increasing the ordering of the CGI fibrils in the direction of mechanical force transmission [83,84]. On the other hand, too high accumulation of GAGs in cartilage and bone tissues leads to progressive cartilage damage, reducing bone growth [86,87].

The primary component of the GAGs fraction in alveolar bone is chondroitin sulphate (ChS) [88,89]. ChS was first extracted from cartilage tissue. However, it is essential to note that interactions between ChS and type II collagen (CGII)—primarily found in cartilaginous tissue—may differ from interactions between ChS and CGI, which predominate in alveolar bone tissue. Although present in lesser amounts in bone than in cartilaginous tissue, ChS indirectly contributes to biomineralisation. Due to its chemical structure, ChS attracts Ca^2+^ and P_i_ ions deposited on collagen fibres to form the bone mineral unit [90,91]. ChS also provides chemical protection; its presence can prevent CGI from acidic denaturation even at low pH levels during inflammation [92]. Furthermore, ChS plays a pivotal role in controlling the shape, size, and number of apatite crystals, promoting the intrafibrillar growth of HA crystals [90,91]. It also enhances bone elasticity, although it may be presumed that this occurs in a manner distinct from that in cartilaginous tissue areas.

#### 2.2.2. Theories of Biomineralisation Mechanism

Given the complexity of the nucleation and growth phenomena of HA crystals, the biomineralisation mechanism still needs to be fully understood. Therefore, two theories are proposed: classical and nonclassical [74].

The classical theory of bone mineralisation suggests that the critical determinant is the occurrence of the substrate, specifically the collagen matrix [74]. Type I collagen (CGI), the most abundant protein in mammals, constitutes 25% (by dry weight) of all proteins. It is one of ten types of collagen, making up about 90 wt.% of all collagens [93]. This protein is expressed in the cells of the skin, ligaments, tendons (fibroblasts), cartilage (odontoblasts), and bone (osteoblasts), serving as the primary structural element in these tissues [94,95].

The nonclassical theory of bone mineralisation posits that this process heavily relies on the activity of osteoblasts and osteoclasts. According to this, the collagen matrix is not the only determining factor for biomineralisation; noncollagen proteins such as bone sialoprotein (BSP), osteocalcin, and SIBLING proteins [53,70,96,97], as well as polysaccharide compounds like glycosaminoglycans (GAGs) [83], are also significant contributors. This theory argues that noncollagen proteins are pivotal in controlling the shape and size of apatite crystals, leading to proper bone mineralisation. Furthermore, the nonclassical theory suggests that mechanical stresses can influence biomineralisation. These stresses potentially modulate the biochemical signal, adding another layer of complexity to our understanding of biomineralisation [98].

Both theoretical viewpoints and the impact of mechanical stress should be considered in hard tissue engineering research designs to provide a comprehensive view of bone mineralisation.

### 2.3. Requirements for Bone Tissue Engineering (BTE)

**Figure 6:**
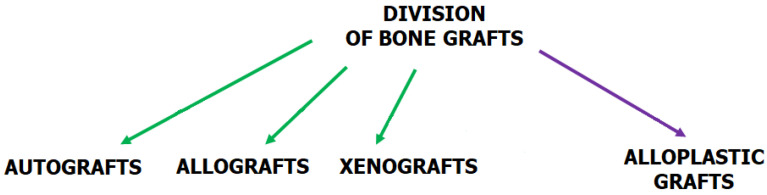
Division of bone grafts according to their origin. An autograft is a tissue transferred from the same individual. It has been considered to be the standard of bone graft replacements. An allograft is a tissue transplanted within the same species, e.g., from one person to another. A xenograft is a tissue or organ that is derived from a species that is different from the recipient of the specimen. An alloplastic graft consists of synthetic biopolymers. It is synthetically derived or made from natural materials [116].

Currently, aliphatic polyester-based scaffolds are gaining popularity over human-origin living bone tissue surgically transplanted (autograft) [99]. Research in this field focuses on composite scaffolds based on PCL, with HA and natural polymers (such as CGI or CS) [100,101,102,103]. In contrast to autografts (see Figure 6), PCL-based scaffolds, supplemented with HA, CGI, and CS, provide more control over the structure, mechanical properties, and composition. For a scaffold to effectively foster the growth of new bone tissue in a defect, it must exhibit properties conducive to bone regeneration. These regenerative properties can be categorised into the following processes: osteoconduction, osteoinduction, osseointegration, and osteogenesis [104,105,106,107].

Osteoconduction is the 3D process of ingrowth of capillaries, perivascular tissue, and osteoprogenitor cells from the bone bed into a structure of the porous implant. The main factor ensuring osteoconductivity is a complex interplay between the microarchitecture and surface of the scaffold and its material type. The microarchitecture (porosity, pore size, and shape) is said to ultimately determine the efficiency of new bone formation and its vascularisation [108]. Osteoconduction is the main driving force of bone regeneration in the case of a scaffold [109].

Osseointegration is defined as a surface (2D) process in which the implant comes into direct contact with the bone and maintains it during functional loading. Its biological measure can be expressed as the percentage of bone in direct contact with the implant or the force needed to remove the implant from the bone. The main factor ensuring successful osseointegration is the determination of the appropriate macroarchitecture of an implant (i.e., its shape and hydrophilicity) [109,110,111,112].

Osteoinduction is a characteristic process inside the scaffold that, via the presence of appropriate biologically active substances (vital proteins and growth factors), encourages the differentiation of mesenchymal stem cells (MSCs) and other osteoprogenitor cells into osteoblasts [83,84]. Moreover, according to Weber, osteoinductive factors are independent of osteoconduction that has occurred [109].

Osteogenesis is the process by which osteoblasts in the scaffold produce minerals that calcify the collagen matrix (biomineralisation), creating a substrate for new bone formation (ossification) [113,114,115].

Bone regeneration is a highly complex process. Oryan et al. assessed the bone repair effect of the (polylactide, PLA)/PCL/HA scaffold without loading it with differentiated bone cells. An in vivo analysis of this scaffold in rats confirmed that the bone tissue’s self-healing potential was not sustained even 80 days postinjury. However, adhesion of native HA particles was observed in several scaffold areas. This result suggests that HA generates a conducive environment for cells to attach and proliferate within the fabricated scaffold [117]. Therefore, a cell-free scaffold can support mild repair effects.

Bone regeneration generally depends on the concurrent occurrence of the healing and ossification processes. Yu et al. studied in vivo the impact of a PCL/HA/CS scaffold loaded with osteogenic markers (e.g., BMP-2) and angiogenic markers (e.g., VEGF, PDGF) on bone regeneration in a rabbit model to achieve a pronounced therapeutic effect. These markers stimulate the differentiation of bone and epithelial tissue, respectively. It appears that both BMP-2 and VEGF mutually enhance the fracture regeneration process [45], which results in a notable improvement in new bone formation. However, regenerating the alveolar bone after tooth extraction remains a significant challenge [118]. Generally, healing and ossification are more cooperative and sequential stages.

In the case of alveolar bone, the inflammatory reaction that starts healing induces significant bone resorption. Although this phenomenon is temporary, it substantially reduces bone volume. Naik et al. conducted clinical trials utilising PCL-based scaffolds inspired by results previously documented in animal studies and performed augmentation of intraoral defects in a selected group of patients [119]. From the drawn conclusions, it can be inferred that the anatomical features of the sinus cavity play a crucial role in the success of local alveolar bone augmentation. New bone growth is observed when the graft material contacts the Schneiderian membranes, the floor, and the walls of the sinus without penetrating the delicate oral mucosa. Moreover, developing a method where the scaffold permits the migration of osteoblasts into the scaffold’s inside has a significant value.

In bone tissue regeneration, numerous biological studies are conducted; for instance, the adhesion, proliferation, and differentiation of osteoblasts in the scaffold are examined. Ultimately, the repair effect of the alveolar bone is determined based on its volumetric growth [120]. Achieving an osteoconductive scaffold with osteoinductive potential involves developing scaffolds with desirable structural, mechanical, degradation, and surface properties to at least induce a mild repair effect on bone tissue. A mild repair effect on bone tissue suggests the potential for the inoculation of cells, markers, and growth factors into the scaffold’s internal environment. Only such a scaffold, combined with cell therapy, results in satisfactory regeneration of bone tissue [27].

Furthermore, combining an osteoinductive strategy with a custom osteoconductive scaffold, identifying the limiting steps of biomineralisation and ossification mechanisms, and comparing the repair effect of the scaffold with a control group (autograft) will provide more reliable results. Despite many constraints, PCL-based composite scaffolds extruded from filament exhibit significant potential for BTE applications [121]. As the electrospun PCL/HA scaffold has been demonstrated to be a robust material for repairing bone defects [122,123] and, when loaded with various drugs and metal particles [124], significantly promoting osteogenic activity, there is a reasonable expectation that the 3D printing method could yield equivalent or even more substantial results [125].

## 3. Material Candidates for Scaffolds

### 3.1. Polycaprolactone (PCL)

**Figure 7:**
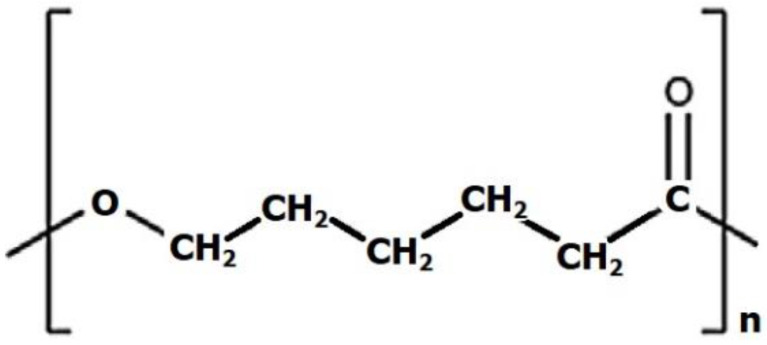
Chemical formula of PCL.

PCL is a linear polyester (Figure 7) characterised by a slow degradation rate, which makes it suitable for potential load-bearing applications [126,127,128]. It has gained approval from the Food and Drug Administration (FDA), enabling new solutions for surgical implant material and drug delivery devices for tissue engineering and regenerative medicine applications [129,130]. Consequently, its usage in fabricating 3D scaffolds for bone engineering has increased. The advantages of PCL include high flexibility and elongation [131], satisfactory biocompatibility, lower acidity of breakdown products compared to other polyesters, and significant load-carrying potential [132].

However, the limitations of pure PCL are worth noting. Due to its poor hydrophilicity, it lacks the osteogenic potential to induce bone regeneration [133]. Therefore, researchers have been combining PCL with various polymers [134,135,136] and inorganic substances [137,138,139] to enhance the biomechanical properties of the scaffolds. The incorporation of bioactive inorganic particles, such as HA [100,103,140,141,142] and CS [143,144,145], into the PCL matrix offers a promising solution to overcome these drawbacks.

### 3.2. Hydroxyapatite (HA)

**Figure 8:**
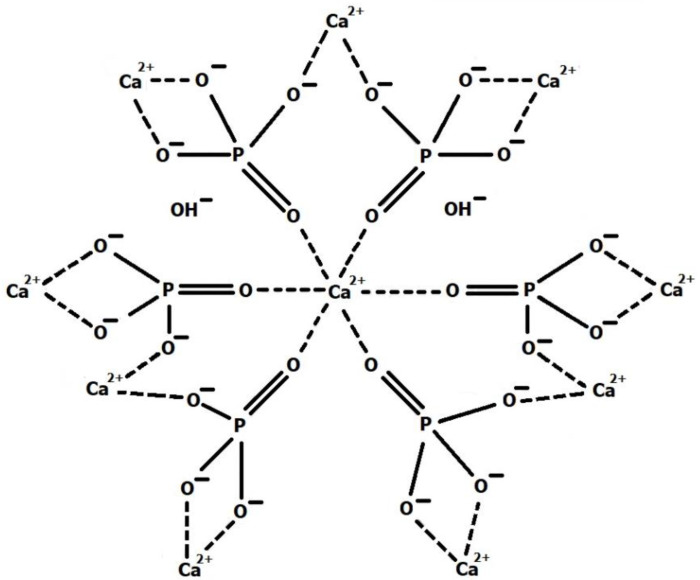
Chemical formula of HA.

The crystalline phases of CaP_i_ exhibit good biocompatibility, which makes them promising materials for repairing hard tissue defects [146]. The most common biomineral CaP_i_ crystal phase is hydroxyapatite ((HA); Ca_10_(PO_4_)_6_(OH)_2_) (Figure 8), which is thermodynamically stable in body fluids [147]. HA has drawn researchers’ interest in recent years due to its porous structure, modification potential, biocompatibility, and bioactivity, the latter referring to its high osteogenic potential [139]. This characteristic greatly accelerates bone and tooth regeneration. Moreover, studies indicate that HA nanoparticles can effectively suppress the growth of various cancer cells [148,149,150]. Over the past decade, HA has gained widespread recognition as a biomedical material used in bone repair, metal implant coating, dental restoration, and drug delivery systems [151].

The source of HA affects its chemical composition, structure, and properties [152]. Numerous methods have been developed to prepare HA. These techniques can be categorised into two groups: (i) biobased extraction methods, and (ii) synthetic methods, which include the hydrothermal method, chemical precipitation method, hydrolysis method, solid-state synthesis method, and sol-gel method [153].

Synthetic HA is the most commonly used material. It is easily obtained from an aqueous solution via a simple coprecipitation method [154]. HA can be produced in various crystallographic forms by reaction between calcium and phosphate salts. A significant feature of HA is its ability to accommodate suitable ionic substitutions, thereby influencing its composition, structure, and solubility. Consequently, ions of similar or smaller size than Ca^2+^ (such as Fe^2+^, Cu^2+^, and Mg^2+^) can be incorporated into the HA crystal lattice with minimal impact on lattice parameters [155].

It was also demonstrated that lanthanides such as Yb and Ho, which have ionic radii much larger than Ca^2+^, can enter the HA crystal lattice without significantly affecting its internal crystal structure [156]. Additionally, the incorporation of Si ions from metasilicate—which is generally absorbed and abundantly distributed in connective tissue—is significantly involved in bone regeneration [157,158]. Biologically synthesised HA created in a physiological environment typically contains various trace elements, including F, Si, Cu, Mg, Sr, Ag, Zn, and Fe, which aid bone regeneration [159,160].

The introduction of necessary elements into the HA structure to enhance its biological activity is appealing due to its compact process and simple composition. However, the conclusions derived from research on HA biomineralisation require further clarification [161]. From an engineering standpoint, the main challenge is to obtain HA with an admixture of a specific element that facilitates a long-term and slow release of trace elements required by the body [162].

Despite its many advantages, introduced HA remains a foreign substance to bone tissue. This is crucial because there are differences in the crystal structure, size, and stability between synthetic HA and in vivo mineralised HA [163]. On the other hand, synthetic HA with the same Ca/P ratio as bone minerals readily converts to bone apatite in vivo. The most limiting feature of HA from an engineering perspective is its high brittleness. To increase the mechanical strength and structure of the final scaffold, many researchers have introduced HA particles into the PCL matrix [117,164]. Ongoing research aims to achieve an evenly distributed high HA content, enabling the body to accept the bone graft material.

### 3.3. Natural Polymers

#### 3.3.1. Type I Collagen (CGI)

One of the challenges in hard tissue engineering is the development of alternative sources of CGI as an initial-stage matrix of biomimetic mineralisation. Currently, CGI is obtained from the skin tissue of cows [165], rat tails [166], and fish [167]. Research is being conducted on using plant collagen in BTE. However, it still requires research and development of technology, in addition to plant collagen and the so-called recombinant collagen. Research is also being carried out to obtain synthetic collagen. This collagen is more accessible to manufacture and can be tailor-made for tissue engineering, but unfortunately, it is not as biocompatible as natural collagen. All of the above sources of CGI have their advantages and limitations. They require further research to provide a safe and effective replacement for natural collagen in bones [168].

**Figure 9:**
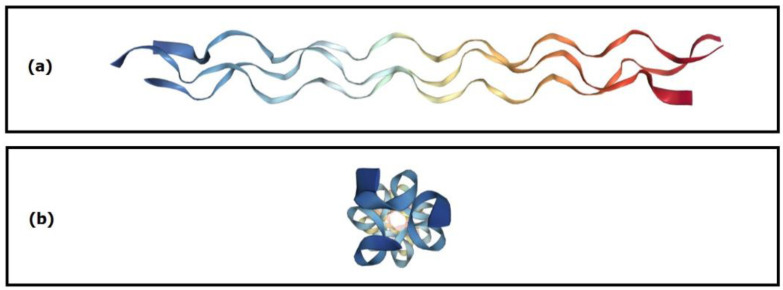
Tertiary structure of CGI. Longitudinal (**a**) and transverse (**b**) view.

CGI, the primary organic component of bone ECM, has a complex structure comprising three hierarchical levels. The base level consists of a triplet of amino acids, primarily proline (Pro), hydroxyproline (Hyp), and glycine (Gly) [169]. These triplets of amino acids are arranged repeatedly to form a secondary structure [170]. The tertiary level is a triple helix formed from three interconnected chains (Figure 9). At the supramolecular level, collagen fibrils form fibres [171], which undergo self-assembly into a hydrogel network under appropriate physicochemical conditions [172].

A systematic analysis of CGI’s primary amino acid sequence reveals a biomacromolecule enriched with charge. Recently, efforts to clarify the role of CGI molecules in mineral nucleation including performing molecular dynamics (MD) simulations [173,174]. Although most MD simulations or simplified in vitro studies neglect complex in vivo biological environments, the significant benefits and relevance of these discoveries and proposed theories are evident. This approach offers the advantage of preventing errors early in research.

Understanding CGI-HA interactions is crucial for comprehending biomineralisation and the formation of bone and tooth tissues [174]. The behaviour of Ca^2+^ and P_i_ ions is influenced not only by the pH and concentration in the environment but also by the type of protein involved, especially its conformation, which dictates the distribution of positive and negative charges on its surface [175].

Wang et al. utilised MD simulation to investigate the initial stage of biomineralisation, characterised by the aggregation and nucleation of Ca^2+^ and P_i_ to form CaP_i_. The results demonstrated that P_i_ ions play a more critical role in biomineralisation than Ca ions. Moreover, the helical conformation of proteins enhances the likelihood of precipitation of the crystalline phase of HA. It is suggested that the intermittent distribution of acidic and basic residues on protein surfaces promotes the formation of large concentrations of Ca^2+^ and P_i_, potentially leading to homogeneous nucleation [175]. The cumulative charge on the protein surface largely dictates the binding affinity of Ca and P_i_ ions [176].

Xue et al. proposed a mechanism for forming CaP_i_ minerals on the surface of CGI, which varies depending on the composition of phosphate and carbonate ions. The presence of HPO_4_^2−^ in the solution is critical for regulating apatite nucleation, while the presence of H_2_PO_4_^−^ inhibits the crystal nucleation process. The inclusion of CO_3_^2−^ in the solution can promote the formation of CaP_i_ clusters. The regulation of apatite clusters can be achieved by altering the ratio of anion concentrations, such as PO_4_^3−^/HPO_4_^2−^ and PO_4_^3−^/CO_3_^2−^. It is hypothesised that mineralisation and demineralisation are strongly tied to the thermal stability of bonds and the kinetics of ion association [177].

As a substitute for CGI, Zhou et al. designed and synthesised a bioactive peptide as a promising agent for preventing tooth decay. It can inhibit demineralisation in an acidic environment and induce self-healing remineralisation in situ [178]. In the outermost layer of the bioactive peptide, positively charged amino acid residues bind to P_i_, while negative charges attract Ca^2+^. Through this strong interaction, HA nucleation sites are formed [179]. Compared to CGI, this protein shows a statistically higher affinity to the tooth surface, stronger counteracting demineralisation, and the promotion of remineralisation of the tooth [180]. The bioactive peptide also has an antibacterial effect. It inhibits the adhesion of *Streptococcus mutans* (cariogenic bacteria) [179].

An integrated experimental and computational approach can provide high-resolution insight into the biomineralisation mechanism. MD simulations combined with empirical studies (e.g., NMR spectroscopy) allow for determining how the peptide interacts with the HA surface [181].

Another way to improve the regulation of HA nucleation processes is through enriched proteins in glutamate and aspartate residues and citrate ions. However, this mechanism still needs to be sufficiently elucidated. Zeng et al. used MD simulations to investigate how citrate ions regulate the adsorption behaviour of polyaspartic acid on the surface of HA in a CaP_i_ solution. Polyaspartic acid can be used as an ion chelate for Ca^2+^ complexation, and it can serve as a template for HA biomineralisation by organizing the distribution of Ca^2+^ on its surface. In mineralisation, citrate ions act as a bridge between the acidic peptide moiety and the HA surface. Thus, the synergistic role of citrate ion and acidic peptide may provide new insight into interfacial phenomena during biomineralisation [182]. In addition to the peptide structure, apatite doping also affects the adhesion of HA to the protein surface. The peptide binds through electrostatic interactions between the cationic peptide molecules and negatively charged groups on the crystal surface [174]. For example, HA modified by Sr^2+^ doping was better at promoting protein adhesion than pure HA [183].

#### 3.3.2. Chitosan (CS)

**Figure 10:**
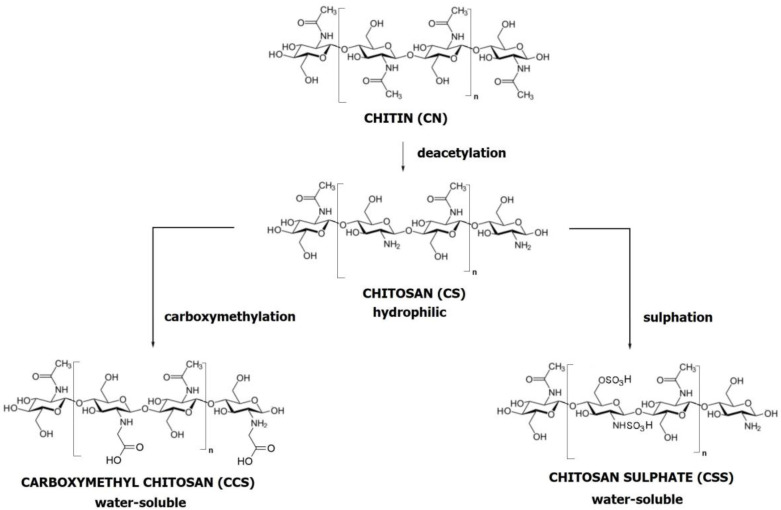
The modification pathways from chitin (CN), through chitosan (CS) to carboxymethyl chitosan (CCS) and chitosan sulphate (CSS).

CS is used in BTE because its chemical structure mimics the biological behaviour of glycosaminoglycans (GAGs) [184,185,186]. CS is a natural linear polymer composed of glucosamine and N-acetylglucosamine units linked by β-1,4-glycosidic bonds with free amino groups. It is the second most naturally occurring polysaccharide. CS is derived from chitin (CN) (see Figure 10), which is isolated from the exoskeletons of shrimps [187], crustaceans, insects, algae, and fungi. To extract CN from, for instance, a crustacean shell, the shell must undergo four steps: pretreatment (washing and drying), demineralisation (acid treatment), deproteinisation (base treatment), and discolouration. CN is then converted to CS through deacetylation (alkaline treatment), which involves the hydrolysis of the acetamide groups and transarrangement of the C-2/C-3 substituent systems in the sugar ring [187,188] (see Figure 10). In general, CS is insoluble in neutral or alkaline solutions due to its specific molecular structure. However, it can dissolve in acidic aqueous solutions (pH < 6.5) by protonating the -NH_2_ moieties [189]. Both CN and CS can form hydrogels due to the large number of functional groups (hydroxyl and amino) available for chemical reactions [190,191]. However, CS has a stronger reactivity and higher hydrophilicity than CN because of the more significant number of amino groups from the deacetylation process [192].

Due to its biological properties (biocompatibility, biodegradability, bioactivity) [193] and polyelectrolyte activity, CS is a suitable organic material for the development of organic–inorganic composite materials, functioning as a carrier or stabiliser of hydroxyapatite (HA) [194,195,196]. Like GAGs, it supports cell adhesion and growth [197,198]. CS also garners attention for its antibacterial properties [199]. Its degradation products are nontoxic, nonimmunogenic, and noncarcinogenic, attributes gaining importance in tissue engineering, wound healing, and drug delivery [200,201,202,203].

The properties of CS are primarily influenced by the degree of deacetylation (DD) and molecular weight (MW). Various DDs and MWs of pure CS can be obtained by secondary treatment of CN [204]. Studies have demonstrated that DD affects the hydrophilicity and biocompatibility of CS, while MW largely determines the degradation rate and mechanical properties. Higher DD correlates with more efficient cell attachment, proliferation, and bioactivity of growth factors. When comparing two types of CS with identical DD but different MW, the one with a higher MW exhibits superior mechanical properties and a slower degradation rate. On the other hand, if two types of CS have the same MW but different DD, the CS with a higher DD degrades more quickly, contrary to the behaviour observed in low-molecular-weight CS [205,206]. Theoretically, CS with higher DD and MW is more suitable for biomedical applications.

CS, acting as a nucleation substrate, impacts the growth mechanism of HA crystals, which can occur in various ways. Depending on the type and content of ECM components, HA can assume different morphologies, such as spheres, rods, whiskers, needles, and plates [207]. MD simulations reveal possible chemical bond formations between the N atoms of CS and the Ca atoms of HA. Furthermore, they indicate the presence of hydrogen bonds between phosphate’s oxygen atoms and CS’s hydroxyl groups. This insight supports understanding the interfacial interaction mechanism, which depends not only on the primary structure of CS but also on the crystal lattice of HA particles. MD simulations predict the behaviour of CS macromolecules on different HA crystallographic planes, indicating that CS macromolecules adsorb more strongly to the HA (1 0 0) surface than to the HA (0 0 1) and HA (1 1 0) surfaces. The interactions between CS and HA were analysed using concentration profiles and pair correlation functions of nitrogen and oxygen atoms in CS [208].

MD simulations are also utilised to understand the mechanism that enhances the mechanical properties of biomaterials intended for hard tissues. Mathesan et al. observed that both Young’s modulus and maximum stress increase with the rising content of HA and the degree of CS cross-linking. Changes in the conformation of CS chains and the evolution of intrinsic structural variables are tied to these mechanical properties. Further results suggest that the formation of hydrogen bonds and electrostatic interactions force changes in various systems [209]. MD simulations enable an understanding of the deformation mechanisms of HA crystals under uniaxial stretching and compression at the nanoscale. Depending on tensile and compressive loading direction, these can lead to significant compression/stretch asymmetry and crystal anisotropy [210]. It is particularly notable as native HA tends to be Ca deficient. In areas of Ca deficiency, cracks form and expand along the direction of the vacancy. While Ca vacancies can impair the mechanical properties of hydroxyapatite [211], it remains an open question whether these cracks in nanocrystals represent the initial stage of a self-healing mechanism (micro-remodelling process) or if they signal the onset of hard tissue damage [212].

The functionalisation of CS is commonly employed to increase bioactivity, mainly focusing on the selective modification of free amino groups [213]. The reactive primary amines and the primary or secondary hydroxyl groups in CS lend it significant versatility [214,215]. CS derivatives are functional across numerous fields, such as food, agriculture, environment, textiles, medicine, and pharmacy. They can be fabricated in various forms, including gels, micro- or nanoparticles, coatings, and scaffolds [216].

A well-known example is chitosan sulphate (CSS) (see Figure 10). CSS mimics the main GAG in alveolar bone: chondroitin sulphate (ChS). CSS is a negatively charged polysaccharide that interacts with positively charged proteins, which could promote osteoblast adhesion, proliferation, or differentiation. The application of nanotechnology is also crucial, as creating structures at the nanoscale level makes it easier to emulate the natural microarchitecture of bone [217,218]. While these mimicry methods cannot fully replicate the complexity and precision of biomineralisation, they can support bone regeneration, especially in its initial stages. Therefore, further research is needed to understand these processes better.

Another example is carboxymethyl chitosan (CMC) (see Figure 10). A CMC-based scaffold coated with tricalcium phosphate (TCP) can biomineralise by forming HA deposits in an ex vivo tooth model [219]. The high mineralisation potential is attributed to carboxyl groups in the organic polymer, which facilitates the spontaneous growth of apatite crystals [220]. The development of these apatite crystals imparts bioactivity to the scaffold and may directly influence its bonding with surrounding structures [221]. Additionally, CMC has antibacterial properties, particularly against cariogenic bacteria, and exhibits considerable potential for wound healing due to its ability to control the expression of the transforming growth factor TGF-β1, interleukin (IL-1), and tumour necrosis factor (TNF-α) [222,223,224].

## 4. Three-Dimensional (3D)-Printed PCL-Based Scaffold

Three-dimensional printing technology has revolutionised BTE by enabling the production of complex architecture PCL-based scaffolds. Moreover, the precision offered by computer-regulated manufacturing in 3D printing allows for optimising control of critical structural properties such as porosity, pore size, and interconnections in bone scaffolds [49,225]. Table 1 lists the key fabrication parameters for PCL-based scaffolds. Manufacturing 3D-printed grafts remains in its preliminary stages, and researchers still need to establish uniform dimensions for these scaffolds dedicated to BTE. Despite this, integrating cutting-edge imaging technologies with 3D printing, such as computed tomography (CT) and magnetic resonance imaging (MRI), enables the designing process of a scaffold architecture for unique tissue defects [13]. As a result, this enhances the efficacy of treating irregular wounds and tissue anomalies, significantly improving medical treatment techniques. In particular, 3D-printed PCL-based scaffolds may significantly contribute to specialised fields such as oral and maxillofacial surgery [11,226] due to their ability to custom-fit unique tissue defects, making them an invaluable tool. Despite the initial high costs associated with research on scaffolds in bone engineering, 3D printing has emerged as a feasible alternative to conventional graft practices [10,227]. Although the high costs associated with the initial stage of research on PCL-based scaffolds in bone engineering are emphasised [119], in the long term, they can reduce the money, time, and effort associated with implantation operations [49]. Consequently, the availability and accessibility of grafts are substantially improved, offering promising prospects for the future of bone engineering.

### 4.1. Processing Conditions

The method of 3D printing for creating bone engineering scaffolds did not develop earlier because solvent casting was the primary fabrication technique. This delay can be attributed to the lack of suitable materials and control methods for extrusion [16,252]. Only recently has research focused on producing dispersions of nanomaterials in polymer matrices, particularly in PCL, which has led to the development of 3D-printed scaffolds in tissue engineering. These processes help meet the requirement for adequate physical connectivity, which is crucial for effective cell anchoring and adhesion [246].

#### 4.1.1. Filament Processing

The extrusion process for PCL filaments necessitates careful consideration of additive content, rheological properties, and processing conditions tailored specifically for the fused filament fabrication (FFF) 3D printing method. Extruded PCL-based filaments, often combined with HA or CS, attract substantial interest due to their osteoinductive potential. Unique behaviour is observed when materials like PCL/HA are used during 3D printing. They form distinctive structures with a higher viscosity than the PCL control sample, requiring increased oversight due to an elevated risk of printer nozzle clogging. Maintaining comprehensive control over the filament deposition temperature and the distance between the filaments is critical to ensuring a successful 3D printing process [16].

Challenges arise when an increase in the HA concentration in the PCL-based filament leads to a corresponding increase in viscosity. This issue can be mitigated by elevating the heating temperature [121,253]. As for material composition, Biscaia et al. argued that incorporating HA into the PCL matrix enhances its mechanical properties, but these enhancements do not proportionally increase with the ceramics’ presence in the composite. The most common weight ratio of polymer to ceramic is 80/20 or 60/40 wt.% [246,254]. In contrast, Rezania et al. suggested that the optimal concentration of HA in the PCL matrix is approximately 30 wt.% [22].

Excessively high HA content and greater fragmentation can increase the risk of ceramic particle agglomeration in the polymer matrix, resulting in a brittle material. However, a proper arrangement of bioceramic reinforcement in the polymer matrix can prevent stress concentration [22,255] and promote the structural integrity of the final material [16,256].

#### 4.1.2. Three-Dimensional (3D) Printing

In the context of processing, PCL is one of the most preferred polymers for extrusion-based 3D printing dedicated to medical applications due to its low melting point of about 60 °C (melting temperature, T_m_) [33,144,257]. Furthermore, the thermal degradation process of PCL does not begin until approximately 300 °C (degradation temperature, T_d_) [34]. This fact indicates high static and dynamic thermal resistance due to the range between T_m_ and T_d_, known as the wide processing window.

From a rheological perspective, molten PCL displays a broad range of viscoelastic behaviours. At lower temperatures within the processing window, it exhibits viscoelastic properties, whereas at higher temperatures, it assumes the characteristics of a viscous liquid. Consequently, the viscosity within this wide processing window can vary during printing. Therefore, adjusting printing parameters (temperature, melt index, printing speed) ensures consistently structured outcomes with similar pore sizes, shapes, and interconnections [121].

Interestingly, Ogata et al. demonstrated that polyesters (e.g., PLA) that have been melted once do not significantly change their viscosity upon reheating at a higher temperature [258]. However, concurrently, Yoshida et al. observed that the polymer’s heating duration affects its extrusion’s smoothness. This effect is likely due to small bubbles in the molten mix escaping during prolonged heating, which results in a more even distribution of extrusion pressure [121].

Moreover, the optimal velocity and temperature printing adjustment relies not only on the polymer features (such as the MW and polydispersity of PCL) but also on the individual 3D printer heating system and variable ambient temperature. Although DSC studies demonstrate the low melting point of PCL (even approx. 51 °C) [259], the optimal temperature for 3D printing PCL from the extruded filament is around 85–90 °C [121]. Therefore, the wide PCL processing window confirms its potential for creating detailed and complex scaffold structures.

Interestingly, Ghorbani et al. [260] observed that the incorporation of HA induces a higher melting temperature and degree of crystallinity in PCL. Conversely, Biscaia et al. noted that incorporating HA into PCL reduces its endothermic melting enthalpy. This effect is likely attributable to the high crystallinity of HA, which is thought to accelerate the nucleation of PCL chain segments, thereby generating a lower degree of PCL crystallinity [246]. Moreover, 3D printing technology promotes oriented crystallisation [261]. The formation of row nuclei facilitates this process, further enhanced by the flow stress applied to the polymer melt during printing [12,261]. Notably, the crystallinity of the polymer significantly influences the mechanical properties of the produced scaffolds [246,248,261].

Lastly, 3D printing technology offers a distinct advantage over conventional manufacturing methods, such as salt leaching [262] and electrospinning [263,264]. As it does not require toxic solvents, 3D printing provides a safer and more environmentally friendly solution.

#### 4.1.3. Hybrid Techniques

Despite its numerous advantages, a 3D-printed PCL-based scaffold has limitations, particularly in the postprocessing stage. The most significant of these are the following: (i) the sterilisation process required before implantation, and (ii) the brittleness of the final scaffold. First, sterilizing PCL-based scaffolds without disturbing their structure can be challenging. This issue partially depends on other characteristics such as mechanical, degradation, surface, and biological properties. Second, some researchers have adopted innovative postprinting modifications like enveloping the 3D-printed PCL scaffold with a layer of CS hydrogel [265] or using a PCL/CS composite [266] hydrogel to develop electrospun sheets, offering a potential solution to the brittleness problem [67]. It is worth noting that CS maintains its thermal stability at PCL printing temperatures. The first melt isothermal process, which occurs above 100 °C, relates more to the loss of bound water than to polysaccharide breakdown [264], thereby preserving its structural integrity during printing. Therefore, to tackle the above problems, 3D printing is combined with other methods, facilitating the development of scaffolds that encapsulate the benefits of both techniques.

3D printing can be combined with electrospinning to produce a scaffold that provides high control over micro- and macrostructure, shape, and mechanical strength. Electrospinning enables the production of fibres with dimensions ranging from microns to the submicron scale. Gonzalez-Pujana et al. demonstrated high metabolic activity and mineralisation of cells cultured on microfibres made from a PCL-based scaffold. Due to electrospun fibres, a microscale structure mimics the cells’ natural environment, promoting osteogenic differentiation [247].

Another hybrid technology involves the combination of 3D printing and freeze-drying. This technology can produce spongy structures with controlled pore size gradients, resulting in scaffolds that exhibit interconnected and adjustable pore structures [267]. When a scaffold is soaked in a solution containing growth factors or other bioactive substances and then subjected to freeze-drying, these bioactive factors become fixed within the scaffold structure [268]. For instance, PCL has been functionalised with a decellularised bone extracellular matrix (dbECM) to produce osteoinductive fibres for 3D printing. Adding bone dbECM to PCL enhanced the mechanical properties of the resulting scaffold, improved cell adhesion, and promoted osteogenesis in mesenchymal stem cells (MSCs) [21,269].

### 4.2. Structural and Mechanical Properties

#### 4.2.1. Size and Geometry of Interconnected Pores

One of the key aspects of scaffolds is their external morphology structure, which affects their performance and tissue interaction. The parameters closely related to the functionality of the scaffold are the size and geometry of pores and their interconnections.

Well-designed porosity with interconnected pores stimulates new bone tissue growth [270]. Additionally, vascular infiltration can be modulated by controlling pore size. Macropores provide space that facilitates cell migration and tissue penetration, thus promoting bone tissue regeneration. Numerous studies have examined the effect of varying pore sizes on the adhesion, proliferation, and migration of different cell types, such as osteoblasts, chondrocytes, and fibroblasts [271]. Oh et al. reported that the appropriate pore size for osteoblast and chondrocyte growth ranges from 300 to 400 μm, while fibroblasts should be around 200 μm [272]. Therefore, the suitable pore size in PCL scaffolds can be estimated to lie between 200 and 400 μm [238]. However, two optimal pore size ranges were also distinguished: 200–600 μm [16,273,274] and 450–700 μm [275]. For instance, Wang et al. used a pore size of 550 μm, which falls within these ranges [16]. Furthermore, pore sizes above 300 μm have been reported to improve vascularity and bone ingrowth [131,246,276,277,278].

Interestingly, Ghayor et al. challenged the long-standing assumption that the optimal pore size for bone scaffolds ranges from 0.3 to 0.5 mm. They contend that the ideal pore size should fall between 0.7 and 1.2 mm. These assertions stem from their work with TPC-based scaffolds produced through 3D printing, which they assessed for potential in facilitating in vivo bone formation. They suggest that these findings may pave the way for innovative methodologies in treating bone defects [36,108].

Building on Ghayor’s paradigm regarding larger pore sizes, the research of Hernandez et al. warrants consideration. They devised a hybrid system using a PCL-based scaffold featuring a pore size of 1.2 mm complemented by a hydrogel. This design exhibited cytocompatibility, as evidenced by the successful adhesion and viability of hMSC within the hydrogel matrix and on the solid scaffold surfaces. Furthermore, biomineralisation tests in SBF highlighted the nucleation and growth of apatite crystals both within the hydrogel and on the PCL scaffold, attesting to its bioactivity. While this hybrid system is optimised for addressing long bone defects, comprehensive in vitro and in vivo studies remain indispensable [230].

Although pore size and geometry are known to influence cell behaviour and tissue formation in vitro, it is unclear how this translates to in vivo scenarios [279]. However, pore architecture inside a scaffold is inevitably necessary for effectively binding growth factors and nutrient transport to cells.

In addition to macropores, scaffolds also contain micropores, which can have positive and negative effects. On the one hand, they can accelerate surface roughness, degradation, and resorption of PCL, which is inherently slow. On the other hand, these micropores could reduce the structure’s compressive strength [246]. Therefore, a balance must be found to maintain the scaffold’s structural integrity.

Equally important is the scaffold’s mechanical behaviour, which heavily depends on a precisely defined pore geometry and directly affects cell interaction. Scaffold structures can be fabricated in various forms, such as helixes, meshes, rings [265], or orthogonal, circular, and sinusoidal shapes [280]. Thus, innovative, versatile, and efficient strategies to 3D print PCL scaffolds with unique anisotropic and curved geometries could better mimic natural tissue.

In conclusion, while size and geometry influence cell behaviour and tissue formation in vitro, our understanding of how this translates to the in vivo scenario remains limited [108,279]. However, one thing is certain: pore architecture is a necessary, though not sufficient, condition for the effective binding of growth factors and transport of nutrients to cells. By appropriately designing porosity, pore size, and geometry, we can achieve scaffolds with optimal mechanical properties, enhancing osteoconductive features.

#### 4.2.2. Mechanical Compressive Strength and Elastic Modulus

In BTE, predicting the forces exerted on native bone during normal function is particularly relevant for designing, fabricating, and integrating a printed scaffold with the host. The most important mechanical property parameters for alveolar bone are the compressive strength and modulus of elasticity, as, during the opening and closing of the jaw, the most significant force induced at the dental symphysis is the compressive force in the transverse direction [281]. The mechanical properties of the 3D-printed PCL scaffold’s layer-by-layer construction can be estimated based on the compact and cancellous bone structure’s volume ratio.

Generally, the compact structure of bone, depending on the orientation of HA crystal growth (transverse or longitudinal), mechanically resembles either a semibrittle or viscoelastic material [282]. The transverse compressive strength of the compact bone structure ranges from 131 to 224 MPa. The modulus of elasticity for compact bone is 17.0 to 20.0 GPa in the longitudinal direction, with a shear modulus of 3.30 GPa and a structural density of 1.80 g/cm^3^ [283].

As for the cancellous bone structure, it is highly porous, and its density is 0.20 g/cm^3^. The compressive strength of the spongy bone structure depends on its apparent density and ranges from 2.0 to 5.0 MPa. The modulus of elasticity ranges from 90.0 to 400.0 MPa [284].

The volume ratio of these structures, constituting the alveolar bone, is determined by various factors such as the location of bone loss in the alveolar bone, age, sex, and health status. The optimal compressive strength might range between 5.0 and 131.0 MPa, while the elastic modulus could lie between 400.0 MPa and 17.0 GPa. Lv et al. contend that scaffolds, which mimic the structure of natural cancellous bone, adapt more adeptly to the microenvironment [285]. Conversely, Pitrolino et al. questioned the validity of designing scaffolds to align with the mechanical properties of native bone tissue. They argued that designing an optimal cellular niche should take precedence over delineating the mechanical strength specifications of scaffolds, as osteocytes—when formed in the appropriate cellular niche—assume the role of structural and mechanical integration within the tissue [286].

Although modelling the alveolar bone’s mechanical properties requires more detailed research and analysis, compact and cancellous bone structures’ compressive strength and elastic modulus provide valuable data. Implanting a scaffold with either excessive or insufficient mechanical strength at the implant site could fail to support native bone growth or lead to bone resorption. Thus, the scaffold’s ability to provide the required mechanical support is a critical criterion for the resulting structure. For instance, Wang et al. obtained PCL-based scaffolds with HA (20 wt.%), achieving compressive strength and elastic modulus of 8.0–11.7 MPa and 11.4–29.2 GPa, respectively. According to the proposal above, these values meet the proposal requirements of the mechanical property of trabecular bones [16].

Apart from orientation, location, and host age, the water environment plays a crucial role in the mechanical assessment of the scaffold. The behaviour of bone in a wet state significantly diverges from that in its dry form [79,287]. To facilitate proper regeneration without notable deformations, the PCL-based scaffold—modified by introducing HA and CS—should offer an elastic modulus identical to that of hard tissues: 1500 MPa in dry and approximately 10 MPa in wet conditions. Pressure causes the liquid to be extruded from the porous structure, thereby increasing the colloidal osmotic pressure and, consequently, the Young’s modulus. An elevated Young’s modulus enhances resilience to high stress [288,289], and the higher the elastic modulus, the less likely the scaffold will deform.

#### 4.2.3. Mechanotransduction

The alveolar bone develops healthily when optimal mechanical forces act on it. Osteocytes embedded in the bone matrix are the primary mechanically sensitive cells involved in the transduction of mechanical stress into a biological response [6]. It is assumed that resorption of the alveolar bone occurs especially when a tooth is missing, i.e., when the alveolar bone is unaffected by mechanical forces. Regarding the designed scaffolds, structural parameters (porosity, pore connections, pore size, and pore geometry) and mechanical parameters (compressive strength and modulus of elasticity) affect how osteocytes perceive mechanical stimuli, which may affect their biochemical reactions. In this respect, the intramembranous ossification of the alveolar bone can be analogously compared to the piezoelectric effect. Since the mechanical stresses in the piezoelectric material generate an electric field (electrical signal), the mechanical stresses in the alveolar bone initiate a biochemical signal towards enzymatic activation for biomineralisation, which ultimately ends in ossification. This unique ability to convert mechanical forces into a biochemical signal is called mechanotransduction. Probably, ionic proteins are responsible for mechanotransduction [290]. These proteins immediately respond to mechanical stimuli by initiating an ionic current. As evidence grows regarding the critical role of this phenomenon in the management of physiological processes, there is a need to identify these proteins [291]. Design scaffolds taking this phenomenon into account become increasingly urgent.

**Figure 11:**
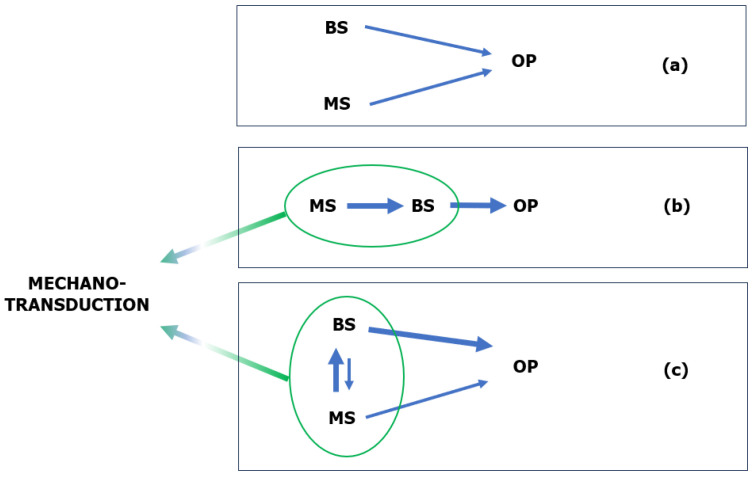
Diagrams of the relationship between mechanical stress (MS), biochemical signals (BS), and ossification process (OP). Several models explaining the relationship between MS and BS in OP may be considered. In the parallel model (**a**), MS and BS are presumed to function independently, yet they concurrently influence OP without mechanotransduction. In the series model (**b**), it is suggested that MS initiates BS, which then triggers OP, indicating the presence of mechanotransduction. Finally, the series-parallel model (**c**) proposes that MS and BS interact with each other through a coupled mechanism, affecting OP, which is characteristic of mechanotransduction. Option (**a**) is a theoretical concept and is regarded as the least likely, while option (**c**), although more complex, is considered the most plausible.

Understanding the relationship between the structural and mechanical parameters of the scaffold and its mechanotransduction capacity is crucial in bone tissue regeneration [122]. The question arises: how does mechanotransduction affect the ossification process? Al-Maslamani et al. developed cell-stretching devices to explore the molecular pathways responsible for cellular responses to mechanobiological processes [292]. Therefore, in the context of BTE, essential factors influencing regenerated bone dynamics are not only biological but also mechanical issues. Figure 11 shows theoretical diagrams of the relationship between mechanical stress (MS), biochemical signals (BS), and the ossification process (OP). Ossification of the healing alveolar bone uses physical movement, mainly compressive forces (less stretching), imitating orthodontic tooth movement.

In a biomechanical context, tensile stress breaks down adjacent ECM molecules, thereby evenly distributing protein molecules. Compressive stress causes the accumulation of adjacent molecules, shortening the distance between HA crystals and affecting bone tissue growth. Both of these stresses regulate bone tissue homeostasis at the local site. Undoubtedly, bone tissue expands as a result of its regular mechanical stimulation. Due to the nature of the load, this stimulation can be divided into two types, static load (Wolf’s law) and dynamic load (Delpech’s law), which affect the formation of bone trabeculae. Too little or too much static load on the bones causes osteoporosis, while too little or too much dynamic load causes atherosclerosis. Disproportionate stress and friction promote inflammation, activating the action of osteoclasts and cytokinins [289]. The hypothesis remains unsettled as to whether the action of compressive and tensile forces in the early period of alveolar process healing is necessary.

### 4.3. Degradation Properties

A particular challenge in designing a PCL-based scaffold is considering its degradation properties. From a materials engineering perspective, degradation deteriorates the structural integrity of the structure and leads to a decline in the mechanical properties of the scaffold. It indicates that a scaffold must be designed so that native bone tissue forms in place of the degraded polymer, subsequently assuming the structural and strength functions. The primary task within this paradigm is to synchronise scaffold degradation kinetics with tissue regeneration rates while maintaining identical structural and mechanical integrity [293]. In the context of bone engineering, PCL has a long degradation time of two to four years [264,294], while bone remodelling takes about six months [295]. Factors that may affect the rate of degradation and the structural and mechanical integration of the scaffold include the following:

- Material characteristics (degree of crystallinity, polydispersity and MW of PCL, amount and type of components with osteoinductive potential in the PCL matrix).
- Topological features (porosity, size and shape of pores, thickness of solid material).
- Degradation environment (pH, ion exchange) [34,296].

#### 4.3.1. Degradation Mechanism

Understanding the degradation mechanism of PCL is essential for its application in the biomedical and pharmaceutical domains [297]. PCL belongs to semicrystalline polymers. Chemical degradation primarily occurs in the amorphous regions of PCL where hydrolysis of ester bonds takes place, breaking longer PCL chains into oligomeric (shorter) ones [296,298,299]. Yoshida et al. reported that the hydrolysis of PCL ester groups exhibits first-order kinetics, meaning that the degradation rate depends mainly on the amount and MW of the polymer [121]. However, an autocatalytic effect emerges in the amorphous regions as the degradation progresses. The cleavage of labile ester moieties, and thus the formation of oligomers with carboxylic and hydroxyl end groups, can uncontrollably accelerate the hydrolysis of adjacent ester moieties [300,301]. PCL degradation products diffuse less easily than other polyesters with shorter aliphatic chains. In addition, the oligomers formed are acidic. Yoshida et al. recorded a pH value that dropped from pH 7.45 to pH 6.11 within 28 days of degradation, a finding also consistent with the downward trend reported by Sung et al. [32]. Lowering the pH confirms that acidic metabolites are formed due to PCL degradation [264]. Although a decrease in pH occurs, PCL degradation products induce fewer pH changes in the local medium than other aliphatic polyesters [296]. Incorporating ceramic particles into a PCL matrix exhibits buffering behaviour [117]. However, a comparison of the degradation kinetics of PCL-based scaffolds containing different ceramic materials still needs to be included [34].

Although the amorphous regions of semicrystalline polymers are more susceptible to hydrolytic degradation [296,299], degradation also occurs in the less accessible crystalline regions, which, in the case of PCL, are represented by numerous spherulites. As the degradation of the intact chains in the crystalline regions progresses, the form of the spherulites changes into so-called “crystalline residues” that take on the structure of “elongated-chain crystallites” [297].

Along with hydrolytic degradation, the phenomenon of recrystallisation occurs. It involves the amorphous areas of PCL rearranging to more ordered structures in contact with the crystalline areas (interspherulitic boundaries). Li et al. reported that adding PCL (approx. 25 wt.%) to other aliphatic polyesters such as PLA (approx. 75 wt.%) leads to an increase in the degree of crystallinity of the composite—from the initial 14 wt.% by mass up to 52 wt.%—after 63 weeks of research on the degradation process. This reorganisation is attributed to the low glass transition temperature of the polymer [302]. The preservation of amorphous regions and the dominance of imperfect and defective crystalline regions helps counteract the phenomenon of recrystallisation, thereby increasing the degradation rate. It can be found that the addition of CS reduces the spherulites and forms more distinct interspherulitic PCL boundaries [260]. The effect of counteracting the phenomenon of recrystallisation is attributed to the hydrolysis of glycosidic bonds between polysaccharide rings in CS chains [265,303], which aids in the hydrolytic degradation of PCL ester groups.

In vivo, enzymatic degradation also occurs, which attacks the crystalline regions of PCL, disintegrating the scaffold structure. In vitro studies in which a PCL-based scaffold was subjected to enzymatic degradation began to be reported in the literature [304,305]. However, Bartnikowski et al. exposed PCL-based 3D scaffolds to concentrated hydrochloric acid (HCl). Acid etching of these scaffolds with different concentrations of HCl, due to the intense penetration of protons (H^+^), ultimately led to the disintegration of the scaffold structure. The features of acid hydrolysis represent degradation under physiological conditions. Acid digestion can be compared to the action of enzymes in the oral cavity at the site of the defect, which, due to inflammation, is locally acidic. By imitating the microenvironment of the cavity, one can estimate material characteristics and scaffold topology, both of which are dependent not only on the structure and components but also on the technique used to manufacture the material [296].

#### 4.3.2. Structural and Mechanical Integrity

The type of erosion determines the structural and mechanical integrity of PCL-based scaffolds. Subject to hydrolytic and enzymatic degradation, scaffolds may undergo either surface or bulk erosion or a combination of both.

Surface erosion is common with polyesters, which, despite being hydrophobic, are highly susceptible to surface hydrolysis. The advantage of surface erosion is that while the structure’s size decreases with weight loss, its density remains unchanged. This characteristic allows the scaffold to maintain its mechanical integrity, a property critical to BTE. Generally, the surface will degrade initially when the scaffold structure comes into contact with a degrading medium. As the medium penetrates the material, most of the scaffold’s solid material begins to break down. This process results in bulk erosion, during which the construct’s volume stays the same, but there is a decrease in density and mechanical strength [306].

Although surface erosion is generally slower than mass erosion, it is typically more desirable because the material decomposes uniformly at a constant rate, making it easier to control. Furthermore, uncontrolled autocatalytic degradation often occurs in conjunction with progressing bulk erosion. It is frequently observed with polyesters, as their acidic degradation products cannot easily diffuse through the polymer network. This results in a localised increase in acidity, which in turn accelerates the scaffold’s degradation in an uncontrolled manner [29,307].

The task involves designing a scaffold with a specific surface erosion timeline and estimating the final stage of degradation when simultaneous surface and volume erosion occurs to predict the time of structural and mechanical disintegration. Maintaining structural and mechanical integrity depends not only on the underlying mechanism of PCL degradation but also on the scaffold’s topology and the surrounding microenvironment [296].

The type of erosion also depends on the fibre thickness and the nature of the solid scaffold material. For instance, once the critical thickness of aliphatic polyester fibre exceeds 2 mm, the degradation mechanism changes from surface erosion to bulk erosion, regardless of ion exchange [306]. In the core part of the materials, partially degraded oligomeric chains accumulate and initiate hydrolysis with a high catalytic effect, potentially leading to accelerated and uncontrolled hydrolytic degradation in the core part—a phenomenon referred to as core-accelerated bulk erosion. However, concerning poly(L-lactide) PLLA, a polyester belonging to the same polymer family as PCL, Tsuji et al. observed that when the diameter of the scaffold fibres is less than 2 mm and optimal ion exchange is ensured, PLLA tends to undergo surface erosion [297]. In this case, ions do not diffuse into the material, meaning that a substantial part remains unaffected. Interestingly, Grizzi et al. noted that foils, powders, and microspheres of PLLA degrade much more slowly than solid material samples [308]. The smaller the polymer size (corresponding to an increased specific surface area), the slower the degradation rate.

Although enhancing the surface area of aliphatic polyester-based scaffold fibres slows down degradation, the degradation process becomes more uniform and easily controlled due to the reduction in internal heterogeneous autocatalytic degradation. An illustrative example is a study on the degradation rate of films of another aliphatic polyester (e.g., PLLA) about porosity. It was found that nonporous PLLA degrades faster than porous PLLA [309,310]. This phenomenon can be attributed to the fact that ion exchange is facilitated in a porous structure, thus inhibiting autocatalytic bulk erosion and maintaining the structure’s integrity for extended periods.

The type of erosion also depends on factors related to the degradation medium, such as pH, temperature, and the presence of ions and enzymes. Therefore, the site of implant placement is vital. In areas with poor vascularisation and low diffusion, degradation products tend to linger, resulting in increased acidity. Bulk erosion is in environments where the ion exchange of Ca^2+^ and P_i_ is restricted [297]. Consequently, the type of erosion is strongly determined by the ion exchange characteristics of the microenvironment. When optimal ion exchange occurs, the types of erosion generally occur sequentially: surface erosion precedes bulk erosion.

In conclusion, the degradation of aliphatic polyesters, e.g., PCL, involves a complex interplay of several parallel and successive phenomena, including water absorption, cleavage of ester bonds, neutralisation of terminal carboxyl groups on the surface, internal autocatalysis, diffusion and solubilisation of oligomers, and recrystallisation effects [311,312]. The type of erosion that PCL naturally undergoes in a physiological environment is currently debated. Although pure PCL scaffolds do not experience significant erosion initially (nominally less than 1 wt.% loss, up to 1 to 3 months) [313,314], the situation alters slightly for a PCL scaffold with incorporated ceramic particles or CS. These scaffolds exhibit weight loss within the first seven days. However, the origin of this material loss is challenging to ascertain. It could be attributed less to PCL degradation and more to the strong binding of water to HA and CS, as these compounds make it difficult to remove moisture [121].

### 4.4. Surface Properties

A scaffold in bone engineering should be biocompatible and biodegradable, and also, its surface should be conducive to migration and anchoring osteoblasts as a direct site of interaction with host cells. The scaffold’s physical properties (e.g., surface roughness, development of the specific surface area) [315] play a critical role in providing cell anchorage [16]. Successful anchoring enables effective cell adhesion [316]. Cell adhesion allows cells to stick to the scaffold’s surface through specific molecular interactions, promotes cell proliferation, and enables cell differentiation through the development of a specific surface area [251,317].

Natural polymers such as proteins (e.g., CGI) and polysaccharides (e.g., CS) are considered suitable materials for facilitating osteoblast anchoring due to the presence of functional groups that differ in polarity, electrostatic charge, and ability to interact via van der Waals forces [318]. Unfortunately, their relatively poor mechanical properties, such as high brittleness and insufficient compressive strength, do not meet the necessary structural and mechanical requirements. Thus, while natural polymers possess high osteoinductive potential, they lack osteoconductive features. This leads to the consideration of synthetic biopolymers like PCL in BTE. PCL has appropriate mechanical properties but is marked by significant hydrophobicity [319]. Modifying a PCL-based scaffold is required to improve its surface wettability [103,320].

One method of enhancing the hydrophilicity of a PCL-based scaffold surface is through the incorporation of HA. The presence of ceramic particles increases the material’s hydrophilicity due to the micropores that appear on the surface of the scaffold fibres, which are associated with greater polarity. While the reasons for the altered surface architecture are unclear, as noted by various authors, a strong correlation has been identified between increased roughness and improved cell adhesion [152,246]. The uniform distribution of HA in the PCL matrix, with some particles exposed on the surface of the filament and some forming agglomerations, amplifies its cellular activity [16,321]. For instance, a higher value of surface roughness of the scaffold fibres was also observed when the concentration of CS was increased [121].

Two specific methods for surface functionalisation, aimed at eliciting desired biological responses, are also noted: gas plasma treatment and the exposure of the polymer surface to other reactive reagents, such as through acid and alkali etching [319].

These methods enhance surface roughness and introduce new functional groups through the covalent modification of the polymer surface due to surface degradation. These surfaces can then be further modified by attaching biologically active compounds such as CGI and growth factors (e.g., BMP-2). This wetting effect can enhance the degree of biomineralisation [322,323,324,325]. The surface properties of PCL can be altered by combining plasma treatment and collagen modification. Studies were also conducted to immobilise collagen on the surface of ultrathin PCL films, significantly improving hydrophilicity after surface modification [326]. The rate of cell attachment and proliferation on the resulting films was increased. Integrins, cytoskeletal and ECM proteins such as CGI and the arginyl-glycyl-aspartic acid (RGD) peptide, can be covalently attached or physically deposited (via electrostatic adsorption) on the PCL surface.

Another method to increase the hydrophilicity of a PCL-based scaffold involves etching with concentrated acids or bases. For instance, a PCL-based scaffold can be immersed in a NaOH solution for 24 h, increasing hydrophilicity promoted by alkaline hydrolysis [238]. This process contributes to the formation of additional carboxylate (COO^−^) and hydroxyl (OH^−^) groups at the ends of the PCL chain on the surface of the scaffold, thereby increasing its roughness through surface erosion. The interaction of the PCL-based scaffold with a drop of water has been found to correlate with scaffold–protein and scaffold–cell interactions [327,328]. Therefore, the contact angle can be interpreted as an indicator of the scaffold’s initial cell adhesion capacity. For instance, PCL scaffolds with higher HA concentrations (20 wt.%) exhibit a smaller contact angle (approx. 78°), reflecting superior hydrophilicity [236].

## 5. Conclusions

Designing scaffolds for alveolar bone augmentation poses a significant challenge. Any discussion to determine scaffold characteristics should begin with a systematic meta-analysis of biomineralisation and ossification. Currently, the literature primarily consists of phenomenological descriptions of these processes. Although our understanding of bone tissue growth at the micro- and macroscopic levels is limited, an examination of the mechanisms of biomineralisation and ossification through the lens of materials engineering and bone tissue can provide direction for the development of osteoconductive scaffolds with osteoinductive potential.

The initial proposal for design involves the selection of materials. A scaffold based on polycaprolactone (PCL), characterised by its optimal biocompatibility, biodegradability, and bioresorbability, emerges as a suitable candidate for scaffolds that exhibit osteoconductive characteristics. Synthetic polyesters can undergo modifications to render their properties suitable for bone tissue engineering (BTE). PCL-based scaffolds, when modified with hydroxyapatite (HA), type I collagen (CGI), and chitosan (CS) particles, demonstrate high osteoinductive potential. Molecular dynamics (MD) simulations of the CGI–HA and CS–HA interactions serve as a guide for in vitro testing.

**Figure 12:**
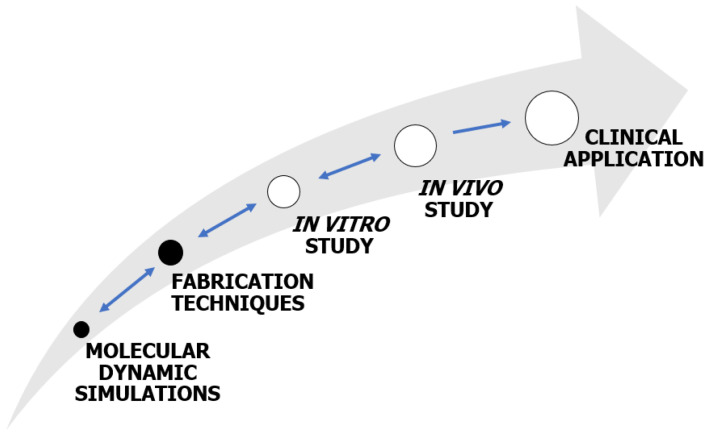
Diagram presenting the pathways from fundamental principles to clinical applications. Each phase of the methodology is predicated on a structured analytical procedure, which entails the decomposition of multifaceted issues into more manageable constituent elements. This is followed by a synthesis procedure, which involves a methodical progression from elementary subjects and phenomena to those of greater complexity. Finally, an enumeration procedure is employed, necessitating a thorough and systematic review to ascertain that all facets of the problem under investigation have been duly considered.

The strategy for designing PCL-based scaffolds is in its nascent stages. Currently, it involves employing heuristic methods to explore the most effective ways of obtaining and analysing scaffold properties. A research path ranging from fundamental principles to clinical applications must be standardised to implement a scaffold in an alveolar bone defect successfully. The present challenge at the meta-analytical level calls for an interdisciplinary approach (Figure 12). Each step of the design process necessitates verifying and evaluating correlation relationships to ensure the scaffold meets the required standards and proves safe and effective for final clinical applications.

Currently, the prevailing trend in designing precise augmentation of alveolar bone defects involves fabricating scaffolds using 3D printing. The ideal pore size and interconnectivity for cell migration, nutrient transport, and vascularisation have yet to be standardised. Moreover, integrating mechanotransduction and cell signals into 3D-printed scaffolds to guide and stimulate bone growth still needs to be explored. The standardisation of mechanical properties is also a subject of debate. Additionally, while most studies initially focus on mechanical properties, the long-term performance of scaffolds in vitro and in vivo, as degradation progresses, remains to be investigated.

Further research is required to determine how to maintain structural and mechanical integrity. Moreover, surface modifications of the scaffold play a pivotal role. A burgeoning area of research involves introducing HA/CS composites into the PCL matrix or depositing CGI onto the surface of the PCL scaffold.

Despite current research gaps, the potential of 3D-printed PCL-based scaffolds reinforced with HA, CGI, and CS for BTE is significant. The design of the scaffolds’ structural, mechanical, degradation, and surface properties could promote osteoblast adhesion, proliferation, and differentiation. These are crucial mechanisms for successful bone regeneration, especially in the case of the alveolar bone, which is sensitive to disturbances in homeostasis. Future research to resolve the discussed limitations will facilitate the development of new concepts and research procedures in BTE.

## Figures and Tables

**Table 1:** Summary of the varied parameters of PCL-based scaffolds.

Composite	3D Printing Method	Scaffold Shape	Scaffold Dimensions (mm)	Interfibre Gap/Pore Size (μm)	Scaffold (Macro) Porosity (%)	Ref.
PCL/β-TCP/CGI	SLS	Cylindrical	12.00(D) × 2.40(H)	300–500	75–77	Liao et al. [17]
PCL/HA, PCL/β-TCP	FDM	Cubic	Mechanical tests: 10.0(L) × 10.0(W)× 10.0(T);	Approx. 800	Approx. 60	Nyberg et al. [228]
		Cylindrical	Other tests: 4.00(D) × 0.64(H)			
PCL/CS	E-jet 3D printing	Cuboidal	21.0 ± 1.7(L) × 12.6 ± 0.9(W) × 123.1 ± 7.2(T)	195.8 ± 9.1	74 ± 3	Wu et al. [229]
PCL (subsequently modified by a CS thermogel)	FDM	Cuboidal	Mechanical tests: 10.0(L) × 10.0(W) × 2.0(T);	325.2 ± 26.3	62.4 ± 0.23	Dong et al. [67]
		Cylindrical	6.0(D) × 2.0(H)			
PCL (subsequently modified by a CS thermogel)	FDM	Cylindrical	15.0(D) × 5.0(H)	Approx. 1200	Approx. 65	Hernandez et al. [230]
PCL/β-TCP	Extrusion-based 3D printing	Cylindrical	8.0(D) × 2.0(H)	Approx. 300		Kim et al. [231]
PCL/HA	SFF	Cubic	5.0(L) × 5.0(W) × 5.0(T)	Approx. 350	Approx. 45	Cho et al. [232]
PCL/TCP	FDM	Cylindrical	6.0(D) × 4.0(H)	Approx. 420		Kurzyk et al. [233]
PCL/GEL/BC/HA	FDM	Rectangular strips	10.0(L) × 5.0(W) × 3.0(T);	Approx. 300	Max. 80	Cakmak et al. [234]
PCL/HA	Extrusion-based 3D printing	Rectangular	Mechanical tests: 10.0(L) × 10.0(W) × 2.5(T);	Approx. 700		Gerdes et al. [122]
			Biological tests: 10.0(L) × 10.0(W) × 2.4(T)			
PCL/PGA	FDM	Disc	Morphological analysis and mechanical tests: 4.0(D) × 4.0(H)	250–400, 150–350, >450	60, 50, 75	Hedayati et al. [235]
		Cuboidal	Biodegradation tests: 10(L) × 6(W) × 2(T)			
PCL/TCP PCL/HA	Laboratory-prepared 3D plotting system	Cylindrical (ring)	3(D_in_) × 8(D_out_) × 3(H)	>200 100–200 <100	Approx. 80	Jeong et al. [236]
		Plate	10(D) × 3(H)			
PCL/HA/HS	FDM	Disc	6.0(D) × 6.5(H)	400	70.8	Liu et al. [237]
			Biological tests: 6.0(D) × 1.0(H)			
PCL (subsequently modified by PEGDA)	FDM	Anatomic	24.7(L) × 11.8(W) × approx. 4(T)	284 ± 31	63	Moura et al. [238]
PCL/PLA/HA (subsequently modified by CS/HS)	FFF	Cylindrical	Mechanical specimen: 6.0(D) × 8.0(H)	500		Thunsiri et al. [239]
		Cubic	Mechanical specimen: 10.0(D) × 10.0(H) Biodegradation specimen: 5.0(L) × 5.0(W) × 5.0(T)			
PCL/HA	Dual-extruder 3D printing	Cuboidal	1.0(L) × 5.0(W) × 5.0(T)	77.1 ± 13.8		El-Habashy et al. [240]
PCL/HA	FFF	Cuboidal	15(L) × 15(W) × 5(T) 15(L) × 15(W) × 3(T) 35(L) × 13(W) × 8(T)	Approx. 1200		Kim et al. [241]
PCL/HA	Cryogenic 3D printing	Orthogonal grid	10(L) × 10(W) × 2(T)	235 ± 43	42.84 ± 1.41	Li et al. [242]
PLA/PCL/HA	Indirect 3D printing	Cubic	5(L) × 5(W) × 5(T)	141.0 ± 47.0	69.00–70.00	Oryan et al. [117]
PCL/HA/SPION	3D printing with a screw-based extruding system	Cubic	5(L) × 5(W) × 5(T)	Approx. 250	45.81	Petretta et al. [243]
		Disc	15.0(D) × 5.0(L)			
PCL/AgNps	FDM	Disc	10.0(D)	431.7 ± 24.6		Radhakrishnan et al. [131]
PCL/CS	3D bioprinting	Cuboidal	10(L) × 10(W) × 5(T)	360	Approx. 55	Rezaei et al. [244]
PCL	3D printing with novel plasma-assisted bioextrusion system	Cuboidal	11(L) × 11(W) × 5(H)	500 1000 1500 2000	60.7 75.5 78.6 85.7	Xu et al. [245]
PCL/CS PCL/β-TPC	3D pneumatic melt extruded scaffold generation	Cuboidal	16.0(L) ×16.0 (W) × approx. 4.0(T)	381 ± 6 395 ± 17	47.0 ± 2.0– 63.0 ± 2.0	Yoshida et al. [121]
			Biological tests: 4.0(L) × 4.0(W) × approx. 2.5(T)			
PCL/HA	3D printing with pneumatic chamber system	Cuboidal	Mechanical tests: 12(L) × 12(W) × 4(T)	Approx. 300		Zimmerling et al. [132]
PCL/HA	3D printing with a screw-fitted chamber system	Cylindrical	10(D) × 2.5(H)	Approx. 350	58.0–60.0	Biscaia et al. [246]
PCL	Combination of 3D printing and electrospinning	Disc	21(D) × 1(H)	Approx. 300		Gonzalez-Pujana et al. [247]
PCL/HA	FFF	Cylinder	Mechanical tests: Approx. 1.6(D) × 80(H)	Approx. 400	37.0	Rezania et al. [22]
PCL/HA/PEGDA	3D printing with extrusion system	Disc	10(D) × 3(H)	380	51.53 ± 2.00 –52.20± 1.67	Sousa et al. [248]
PCL/HA	FFF	Cuboid	Morphological analysis: 15(L) × 15(D) × 2(H) Mechanical tests: 15(L) × 15(D) × 4(H)	Approx. 550	60.0 ± 0.9 –65.4 ± 0.3	Wang et al. [16]
PCL/HA PCL/TCP	FDM	Cuboid	31.0(L) × 26.7(D) × 10.0(H)	Gradient of approx. 200, 300 and 500, 600	Approx. 52.0	Daskalakis et al. [34]
PCL	SLS	Disc	20(D) × 2(H)	Approx. 500, and approx. 700	49.7 ± 3.0–63.6 ± 3.0	Janmohammadi et al. [18]
PCL/CS (subsequently loaded with VAN)	FDM	Disc-shaped	8.00(D) × 1.50(H)	Approx. 200	Approx. 59.0	López-González et al. [249]
PCL/CS	RP	Anatomic (mandibular condyle)		100–200		Lee et al. [250]
PCL/MMBG/Al_2_O_3_	LDM	Cubic	5(L) × 5(W) × 5(T)	0.005–0.020 (mesopores)	Above 80	Najafabadi et al. [251]

Abbreviation: PCL: polycaprolactone; TCP: tricalcium phosphate; HA: hydroxyapatite; HS: heparan sulphate; MMBG: magnetic mesoporous bioactive glass; AgNps: silver nanoparticles; PEGDA: diacrylate poly(ethylene glycol); VAN: vancomycin; RP: rapid prototyping; SLS: selective laser sintering; FDM: fused deposition modelling; LDM: liquid deposition modelling; FFF: fused filament fabrication; SFF: solid freeform; L: length; W: width; T: thickness; D: diameter; D_in_: inner diameter; D_out_: outer diameter.

## Data Availability

No new data were created.
